# A Breach in the Lung: Broncho-Pleural Fistula in the Setting of Granulomatosis With Polyangiitis

**DOI:** 10.7759/cureus.102662

**Published:** 2026-01-30

**Authors:** Saeed R Mohammed, Rei S Medford, Steven Sankar, Ravindra Basdeo, Jessica Rampersad, Narine Mack

**Affiliations:** 1 Medicine, Medical Associates Hospital, St Joseph, TTO; 2 Medicine, Independent Research, San Fernando, TTO; 3 Radiology, San Fernando General Hospital, San Fernando, TTO; 4 Medicine, San Fernando General Hospital, San Fernando, TTO

**Keywords:** bronchopleural fistula, chronic rhinitis, granulomatosis with polyangiitis (gpa), pneumothorax (ptx), spontaneous pneumothorax

## Abstract

A 16-year-old female presented with complaints of a persistent cough for 2 weeks, productive of yellow sputum. She reported subjective low-grade fever for 4 days prior to presenting, with a prior 6-week history of nasal congestion, frontal headaches, and facial pain. ENT review revealed hypertrophy of the right nasal turbinates with features of chronic rhinitis with grossly normal nasal flexiscopy.

Imaging revealed focal areas of consolidation in the left lung, with a cavitating lesion. Investigations revealed a leucocytosis, elevated erythrocyte sedimentation rate (ESR), and an elevated proteinase 3-specific antineutrophil cytoplasmic antibodies (cytoplasmic antineutrophil cytoplasmic antibodies) (PR3-ANCA (c-ANCA)). Granulomatosis with polyangiitis (GPA) was suspected, prompting commencement of methylprednisolone 1 g intravenously for three days, then oral prednisone at 1 mg/kg. Five days later, the patient developed a left pneumothorax with partial collapse of the left lower lobe and a likely bronchopleural fistula. Further clinical deterioration led to the patient's transfer to the thoracic surgical unit at another institution.

We recommend that clinicians be cognizant of the potential for spontaneous pneumothorax among patients with GPA, especially in the presence of large cavitary nodules. New-onset or worsening chest pain or dyspnoea should prompt further evaluation, and patients must be closely monitored whilst on immunosuppressive therapy.

## Introduction

Granulomatosis with polyangiitis (GPA) is a multisystemic necrotizing vasculitis primarily affecting the small and medium vessels and a member of the anti-neutrophil cytoplasmic antibody (ANCA) associated vasculitis group [1]. The upper respiratory tract, lungs, and kidneys are the most commonly affected organs, but the manifestations are heterogeneous, ranging from asymptomatic to fulminant multiorgan vasculitis leading to death [1]. Diagnosis of GPA is based on a combination of clinical features, imaging appearances, laboratory assessment of inflammatory and serologic markers, and histological evaluation [1,2].

Chest radiographs demonstrate abnormalities in most patients with GPA, typically pulmonary nodules or consolidations [3]. Computed tomography (CT) imaging has greater sensitivity and specificity [4] and frequently reveals nodules, consolidation, masses, ground-glass opacities, and bronchial wall thickening [4,5]. Such radiological findings correlate with disease activity and show response to treatment [4,5]. Pulmonary nodules appear in 70-90% of cases [4-6] and may be single or multiple, affecting one or both lungs. Cavitating lesions are observed in 10-50% of patients [1,4,5], and may cause coughing or haemoptysis [1]. Spontaneous pneumothorax is a rare but recognized complication with an estimated incidence of 3-5%, albeit not often reported in the literature [7]. The underlying pathophysiology is uncertain and likely multifactorial, with postulated mechanisms including breakdown of a subpleural cavity nodule and subsequent bronchopleural fistula and complications of immunosuppressant therapy and infections [7,8].

We here present the case of a teenage female patient who developed a pneumothorax, bronchopleural fistula, and further thoracic complications shortly after commencing therapy for newly diagnosed GPA.

## Case presentation

A 16-year-old female presented with complaints of a persistent cough for 2 weeks, productive of yellow sputum. She reported subjective low-grade fever for four days prior to presenting.

She related a 6-week history of nasal congestion, associated with frontal headaches and facial pain. She presented to the Otolaryngology department 5 weeks prior for an assessment. CT paranasal sinuses had demonstrated hypertrophy of the right nasal turbinates with features of chronic rhinitis, whilst a nasal flexiscopy was grossly normal.

She noted a hoarse voice for the past several weeks but denied arthralgia, hair loss, stomatitis, skin changes, or urinary symptoms. Her cough was associated with pleuritic chest pain and dyspnoea on exertion without haemoptysis. There was no history of ill contacts or recent travel.

**Figure 1:**
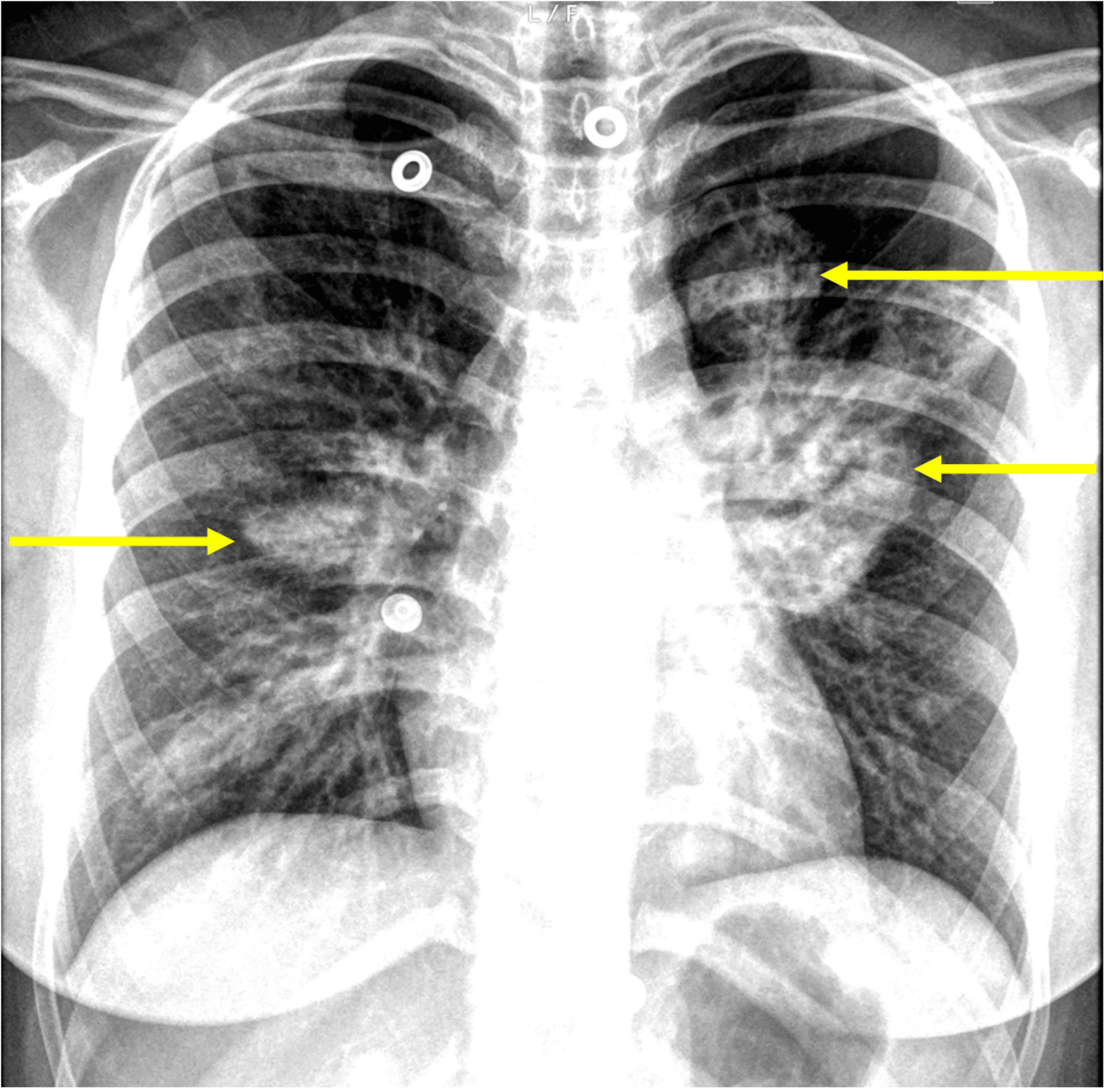
Formal PA Chest Radiograph Formal PA Chest Radiograph illustrating large well-circumscribed opacified regions (highlighted with yellow arrows) within the peri-hilar regions as well as within the left upper lobe. There are subtle, smaller regions of ill-defined opacifications seen at the lower lung zones. PA:  posteroanterior

**Figure 2:**
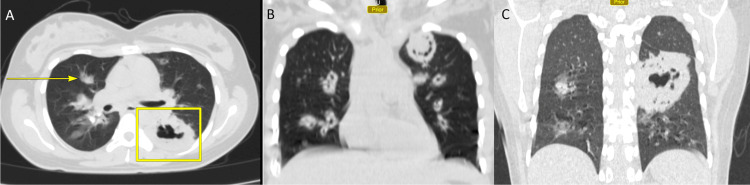
Axial CT Chest Pulmonary Window Axial CT chest pulmonary window illustrating patchy opacities (yellow arrow) with a large cavitating lesion within the superior segment of the left lower lobe in Panel A. Coronal CT Chest pulmonary window illustrating multiple bilateral thick-walled cavitating lesions in Panel B, with the largest cavitating lesion within the superior segment of the left lower lobe in Panel C measuring 8.0 cm in diameter.

Chest radiography (CXR) revealed large, well-circumscribed regions within the perihilar regions as well as within the left upper lobe (Figure 1), prompting a CT chest, which displayed focal areas of consolidation in the left lung, with a cavitating lesion (Figure 2). 

Investigations revealed a leucocytosis, elevated erythrocyte sedimentation rate (ESR), and an elevated proteinase 3-specific antineutrophil cytoplasmic antibodies (PR3-ANCA). HIV and *Mycobacterium tuberculosis *interferon gamma release assay (IGRA) testing were negative, whilst anti-glomerular basement membrane (anti-GBM), perinuclear anti-neutrophil cytoplasmic antibody (P-ANCA), urinalysis, and renal function were within normal limits (Table 1). A biopsy with subsequent histopathological investigation was not performed due to limited facility and resource availability at the time. 

**Table 1:** Laboratory Investigation Results PR3-ANCA/C-ANCA: proteinase 3-specific anti-neutrophil cytoplasmic antibodies/cytoplasmic anti-neutrophil cytoplasmic antibodies; P-ANCA: perinuclear anti-neutrophil cytoplasmic antibodies; ANA/ANF: antinuclear antibody/antinuclear factor; Anti-GBM ABS: anti-glomerular basement membrane antibodies; IGRA: interferon gamma release assay; n/a: not applicable

Investigation	Result	Units	Reference Range
WBC Count	13	10^3^ / µL	4.8 – 11
ESR	94 mm/hr	mm/hr	0 – 20
Creatinine	0.5	mg/dL	0.5 – 1.0
Blood Urea Nitrogen	5.0	mg/dL	5.0 – 23.0
Sodium	136	mmol/L	135 – 145
Potassium	4.5	mmol/L	3.5 – 5.1
Chloride	98	mmol/L	97 – 110.0
PR3-ANCA (c-ANCA)	131	n/a	0 - 5 NEGATIVE| 6 - 10 BORDERLINE | 11 - 50 POSITIVE | >50 STRONG POSITIVE
P-ANCA	1	n/a	0 - 5 NEGATIVE| 6 - 10 BORDERLINE | 11 - 50 POSITIVE | >50 STRONG POSITIVE
Anti-GBM ABS	5	n/a	0 - 5 NEGATIVE| 6 - 10 BORDERLINE | 11 - 50 POSITIVE | >50 STRONG POSITIVE
ANA/ANF 1/100 Dilution	Negative	n/a	n/a
ANA/ANF Pattern	Negative	n/a	n/a
HIV	Negative	n/a	n/a
*Mycobacterium tuberculosis* IGRA	Negative	n/a	n/a

**Figure 3:**
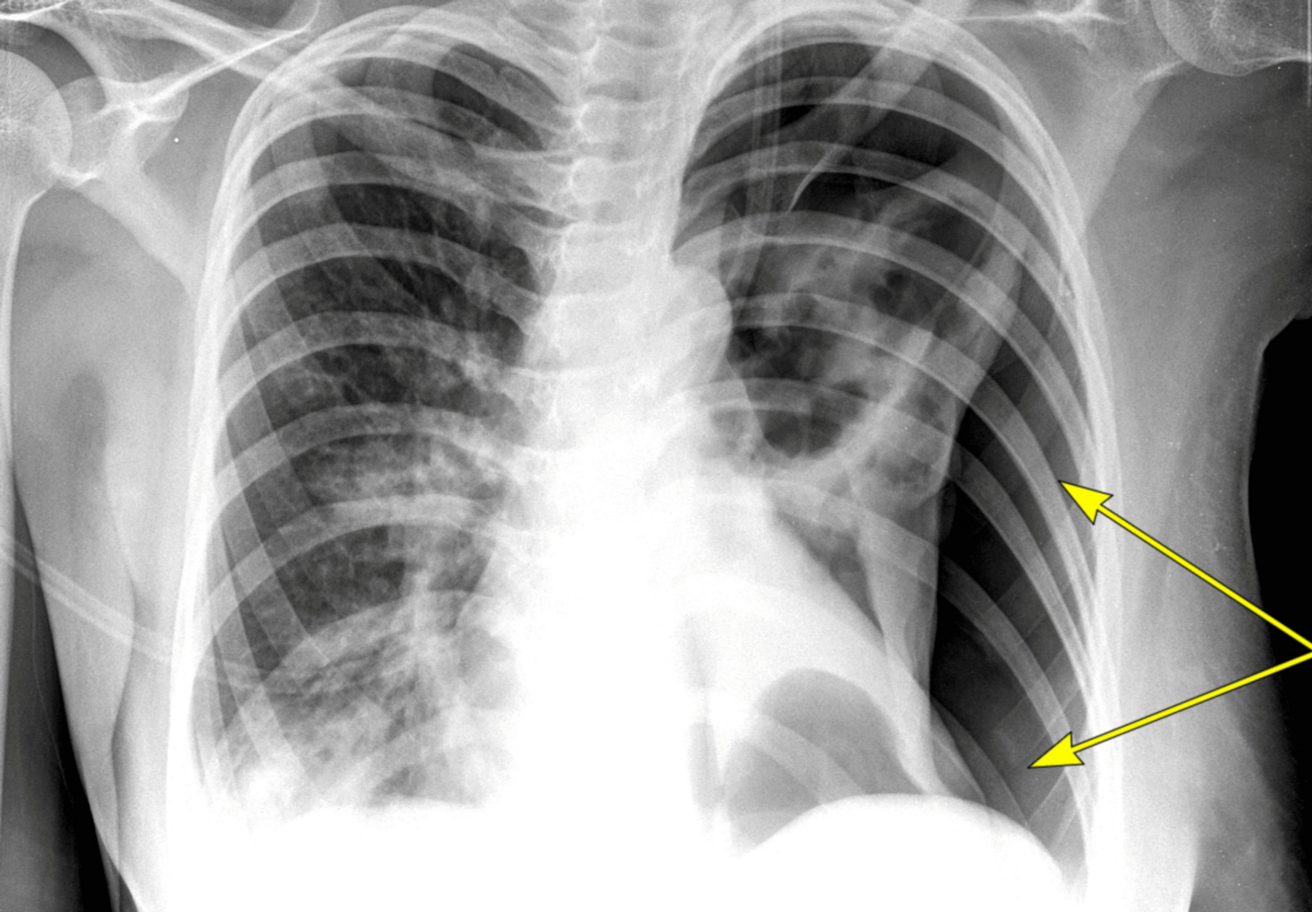
Portable Chest Radiograph Portable chest radiograph illustrating a large radiolucent region along the lateral aspect of the left hemithorax with no mediastinal shift noted. No pleural effusion is present.

**Figure 4:**
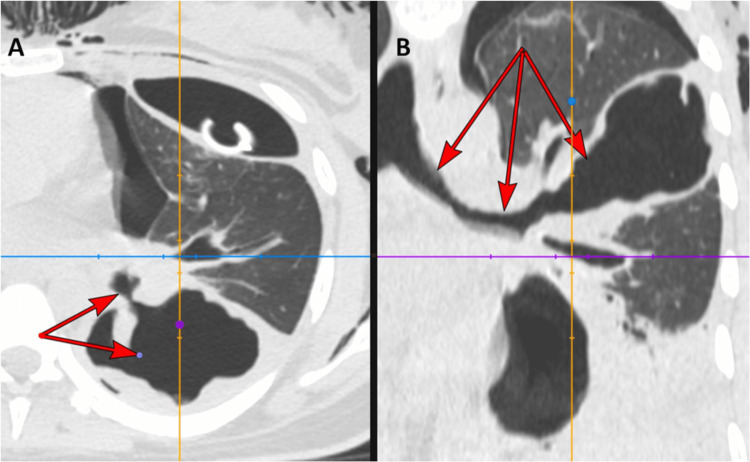
CT Chest In Panel A, axial pulmonary-window imaging demonstrates a high-flow fistulous communication (red arrows) extending from the left main bronchus to the pleura. This finding is also shown on the coronal view in Panel B, which also illustrates the surrounding parenchymal destruction.

Granulomatosis with polyangiitis (GPA) was suspected, with clinical, radiological, and laboratory investigations producing a combined score of 11 as per the 2022 American College of Rheumatology/European Alliance of Associations for Rheumatology (ACR/EULAR) criteria for GPA. Methylprednisolone 1 g intravenously was commenced for three days, then oral prednisone at 1 mg/kg. Five days later, the patient reported worsening dyspnoea, peripheral oxygen saturation (SpO2) on room air = 89%. Chest X-ray revealed a left pneumothorax (Figure 3), with repeat CT chest confirming pneumothorax with partial collapse of the left lobe and a likely bronchopleural fistula (Figure 4). An intercostal chest tube was inserted. Sputum culture and pleural fluid analysis both demonstrated *Pseudomonas aeruginosa*, and thus meropenem was initiated. 

The patient continued to deteriorate clinically, and CT pulmonary angiography revealed pneumopericardium and pneumomediastinum, leading to her transfer to the thoracic surgical unit at another institution.

## Discussion

The patient’s diagnosis of GPA was made as per the American College of Rheumatology’s 2022 criteria [1]. Cavitating nodules and ground-glass opacities are well-documented features of GPA. Allen and Harvey [9] in their pictorial review of 155 patients with GPA concluded that nodular masses >2 cm cavitate in at least 25% of cases, whilst Martinez et al [4] concurred that central cavitation was more common in nodules larger than 2 cm.

Shi et al [8], in their 2018 analysis of 25 reported cases of pneumothorax secondary to GPA, found that these patients more frequently had cavitating pulmonary nodules than those in the general GPA population. Further, secondary infection (often with the same pathogen identified in sputum and pleural fluid drainage) was likely associated with cavity formation, whilst immunosuppressants increased the risk of infection and delayed wound healing [8]. Bronchial pleural fistula was deemed a pathological feature for pneumothorax [8]. Glucocorticoid therapy has been deemed a major modifiable cause of adverse events in patients with ANCA-associated vasculitis, particularly during the induction period [10].

Pneumomediastinum and pneumopericardium are rare manifestations of GPA, described in only a small number of cases [11-13]. Alhazmi et al [13] have postulated a pathological pathway for pneumomediastinum in this clinical context, starting from mucosal necrosis secondary to GPA vasculitis and then rupture of alveolar connective tissue. Siosi et al [11], in their description of a case of pneumomediastinum in a woman with GPA, reported no structural source of air leak and concluded that GPA was the underlying cause.

Whilst 5-10% of GPA cases include cardiac involvement [6], typically pericarditis and coronary vasculitis, pneumopericardium is not a well-recognized manifestation. The patient reported herein had extensive cavitating disease, a superimposed *Pseudomonas *infection, and early high-dose corticosteroid exposure. Thus, her clinical deterioration, by way of spontaneous pneumothorax and air-leak syndromes, is likely the result of a culmination of risk factors. Clinicians should maintain a high index of suspicion for pneumothorax and air-leak syndromes, and new respiratory symptoms/decline should warrant early repeat imaging. Early thoracic surgical consultation should be sought, if available.

## Conclusions

We report the case of an adolescent patient with granulomatosis with polyangiitis who developed spontaneous pneumothorax shortly after commencement of steroid therapy, then pneumopericardium and pneumomediastinum.

We recommend that clinicians be cognizant of the potential for severe pleural manifestations of GPA, especially in the presence of large cavitary nodules. New-onset or worsening chest pain or dyspnoea should prompt further evaluation, and patients must be closely monitored whilst on immunosuppressive therapy.
